# Short Duration Combined Mild Hypothermia Improves Resuscitation Outcomes in a Porcine Model of Prolonged Cardiac Arrest

**DOI:** 10.1155/2015/279192

**Published:** 2015-10-08

**Authors:** Tao Yu, Zhengfei Yang, Heng Li, Youde Ding, Zitong Huang, Yongqin Li

**Affiliations:** ^1^Emergency Department, Sun Yat-sen Memorial Hospital, Sun Yat-sen University, Guangzhou 510120, China; ^2^Institute of Cardiopulmonary Cerebral Resuscitation, Sun Yat-sen University, Guangzhou 510120, China; ^3^Emergency Department, Affiliated Dongguan Hospital, Medical College of Jinan University, Dongguan 523900, China; ^4^Biomedical Engineering Department, Guangzhou Medical University, Guangzhou 511436, China; ^5^School of Biomedical Engineering, Third Military Medical University, Chongqing 400038, China

## Abstract

*Objective.* In this study, our aim was to investigate the effects of combined hypothermia with short duration maintenance on the resuscitation outcomes in a porcine model of ventricular fibrillation (VF).* Methods.* Fourteen porcine models were electrically induced with VF and untreated for 11 mins. All animals were successfully resuscitated manually and then randomized into two groups: combined mild hypothermia (CH group) and normothermia group (NT group). A combined hypothermia of ice cold saline infusion and surface cooling was implemented in the animals of the CH group and maintained for 4 hours. The survival outcomes and neurological function were evaluated every 24 hours until a maximum of 96 hours. Neuron apoptosis in hippocampus was analyzed.* Results.* There were no significant differences in baseline physiologies and primary resuscitation outcomes between both groups. Obvious improvements of cardiac output were observed in the CH group at 120, 180, and 240 mins following resuscitation. The animals demonstrated better survival at 96 hours in the CH group when compared to the NT group. In comparison with the NT group, favorable neurological functions were observed in the CH group.* Conclusion.* Short duration combined cooling initiated after resuscitation improves survival and neurological outcomes in a porcine model of prolonged VF.

## 1. Introduction

Out-of-hospital cardiac arrest (OHCA) continues to be a major health problem around the world. There are more than 800,000 victims in western society and 540,000 victims in China each year [1, 2]. Most of the patients resuscitated successfully suffer from postcardiac arrest syndrome (PCAS) and do not leave hospitals with positive neurological outcomes. Hypothermia is a proven useful therapy to improve the neurological and survival outcomes after cardiac arrest and resuscitation [3, 4]. For the patients who are still comatose after the return of spontaneous circulation (ROSC), the guidelines recommend access to mild therapeutic hypothermia (MTH) to reduce neurological deficit. In clinical settings, target temperature management is widely applicable when the core temperature is maintained within 32°C–34°C for 12–24 hours [5, 6]. Still, the optimal duration of MTH is uncertain.

In animal studies, intra-arrest MTH is considered to have better survival and neurological recovery [7]. When MTH is initiated earlier, larger benefits are obtained. Our previous study demonstrated that intravenous infusion of cold saline (ICS) was not the correct choice as an intra-arrest cooling method [8]. ICS is not an ideal method to maintain target temperature and an excessive volume load may compromise the resuscitation outcomes [9, 10]. However, ICS is an attractive strategy to achieve early cooling after resuscitation when compared with surface cooling. ICS is easy to administrate and has a shorter induction period [3, 9, 11]. Therefore, in-time limited volume ICS combined with surface cooling might be a better cooling strategy following resuscitation.

To achieve enough protective effects, prolonged MTH was adopted worldwide in clinical practice [3, 5, 6]. Long-time cooling increases the risks of hypothermia-related complications, such as electrocardial instability, hemodynamic imbalance, and electrolyte abnormalities [12]. These complications might eventually impair the advantages of hypothermia for survivors. In a rat study by Ye et al., the authors demonstrated that neurological prognosis was associated with the duration of target temperature management. To achieve additional benefits, the cooling duration should be less than 2 hours [13].

We hypothesized that a combined cooling method initiated immediately after ROSC with volume limited ICS and surface cooling might yield better survival and neurological outcomes with shorter cooling duration. In this study, we sought to investigate whether a shorter duration of combined cooling would improve the resuscitation outcomes in a porcine model of prolonged cardiac arrest and resuscitation.

## 2. Materials and Methods

### 2.1. Study Design

The controlled and prospective animal experiment was designed to test the hypothesis that a shorter duration of mild hypothermia initiated earlier and after ROSC can improve the outcome in a porcine model of CPR. All animals received humane care. The experimental protocol was approved by the* Institutional Animal Care and Use Committee of Sun Yat-sen University (IACUC-2012-0801).*

### 2.2. Animal Preparation

Fourteen male domestic pigs, weighting 34–36 kg, were fasted overnight with the exception of free access to water. Ketamine (20 mg/kg) was intramuscularly injected to induce anesthesia. Sodium pentobarbital (30 mg/kg) was given intravenously via an ear vein to block spontaneous respiration and maintain anesthesia (8 mg/kg, after ROSC every hour). A 7-F curve cannula was advanced into the trachea to establish artificial airway. The location was confirmed by an infrared gas analyzer (Model 1265, Medical SYSTEMS Inc., CT, USA). The animals were then mechanically ventilated with a total volume of 15 mL/kg body weight, fraction of inspired oxygen (FiO_2_) 0.21, a respiration rate of 12–20 breaths/min, and maintained PetCO_2_ at 35–40 mmHg.

Animal preparation was followed as stated in our previous study [14]. In brief, to obtain arterial pressure (AP), a 6-F catheter (Cordis brite tip GC, Bridgewater, NJ, USA) was advanced from the right femoral artery, approximately 45–50 centimeters, to the level of the descending aorta. To measure the right atrium pressure (RAP) and the core blood temperature (*T*_c_), a 7-F four-chambered Swan-Ganz catheter (774HF75 Swan-Ganz TD Cather, Edwards Lifesciences Corporation, Irvine, CA, USA) was advanced from the right femoral vein and floated to the pulmonary artery with the assistance of a characteristic arterial waveform. For the measurement of the brain temperature (*T*_b_), a 5Fr Swan-Ganz catheter (Arrow International, Inc., Reading, PA, USA) was retrograded 5–7 cm and advanced into the cranial cavity from the internal jugular vein. To measure the left ventricle pressure (LVP), a 6-F catheter (Cordis brite tip GC, Bridgewater, NJ, USA) was advanced into the left ventricle based on the feature of pressure waveform. The hard gel types of adult defibrillation pads (stat-padz, Zoll Medical Corporation, Chelmsford, MA, USA) were applied with an anterior to lateral placement. To measure the compression depth, an accelerometer-based handheld CPR device (CPR-D-padz, Zoll Medical Corporation, Chelmsford, MA, USA) was placed on the surface of the porcine's sternum and underneath the rescuer's hands during chest compressions. Arterial blood samples were measured via a handheld blood-gas analyzer (Model CG4 + Cartridge, Abbott i-STAT System, Princeton, NJ, USA). Cardiac output was measured by the thermodilution technique with the aid of a cardiac output computer that was described in a previous study (Baxter COM-2, Edwards Division, Santa Ana, CA, USA) [14].

### 2.3. Experimental Procedure

To electrically induce VF, a 5-Fr pacing catheter (Cordis brite tip GC, Bridgewater, NJ, USA), guided with an endocardial electrocardiogram via a multiple parameter monitor (78352C, HP Corporation, Palo Alto, CA, USA), was advanced from the right external jugular vein into the right ventricle. A 2 mA alternating current was delivered to the endocardium of the right ventricle within 5 secs and maintained untreated VF for 11 minutes.

Two researchers initiated a two-person CPR algorithm of adult basic life support as recommended by the 2010 guidelines of the* America Heart Association*. Rescuers provided high-quality CPR with at least 100 compressions per minute, allowing complete chest recoil and minimum interruption. Compression depths were approximately 25% of thoracic anteroposteral diameter. Room air was delivered with the bag device with a ratio of 30 chest compressions to two ventilations. Two minutes after CPR, a bolus of epinephrine (30 *μ*g/kg) was administrated into the right atrium. Six minutes following CPR, a single 120-J biphasic shock (M-Series, Zoll Medical Corporation, Chelmsford, MA, USA) was attempted to terminate VF. The animals that achieved ROSC were randomized into the following two groups: control group (NT) and combined hypothermic group (CH). In the NT group, *T*_c_ was maintained at 37.5–38.5°C until 6 hours after ROSC and received room temperature saline (25°C–28°C, total volume is 30 mL/kg) via superior vena cava by the use of a standard infusion set and a pressure bag inflated to 300 mm Hg as soon as ROSC was obtained for 30 mins. In the CH group, ice-cold saline (4°C, total volume is 30 mL/kg) was infused steadily by the same method as the NT group. Intravenous hypothermia was maintained in the first 30 mins after ROSC in order to induce immediate targeted mild hypothermia. Meanwhile, surface cooling with a water blanket (HGT-200II, Hokai Medical Instruments Corporation, Zhuhai, China) was activated to induce and maintain mild hypothermia (target temperature: core temperature is maintained at 32°C to 34°C). In our hypothermia protocol, combined cooling was induced and maintained at the target temperature for 4 hours after ROSC. The animals were actively rewarmed during the last 2 hours of the experiment to the normal temperature (38°C). All animals were treated equally throughout the study with the exception of the temperature management. The animals were allowed free access to food and water and were observed for an additional 96 hours.

### 2.4. Measurement

Baseline measurement was obtained, including aortic pressure, cardiac output, blood analysis, and pulmonary core temperature. The ECG, pressure measurements, and acceleration signals were continuously measured and recorded through a data acquisition system supported by Windaq hardware/software (Dataq Instruments Inc., Akron, OH, USA) at a sample rate of 300 Hz. The coronary perfusion pressure (CPP) was digitally computed from the differences in time-coincident diastolic aortic and right atrial pressures. The compression rate and depth were calculated from the double integration of acceleration signals recorded from accelerometer by Matlab 7.0 (The Math Works, Inc., Natick, MA, USA). The cerebral performance category (CPC) and neurological deflect score (NDS) were used to evaluate neurological function every 24 hours by two independent researchers [8]. NDS is a method of semiquantitative analysis for ischemia neurological deficit in three levels of consciousness, motor, and sensory function and behavior. A higher score usually indicated severe injury. A score of 400 equals death and a zero indicates normal function. CPC is an alternative scale from the view of consciousness and behavior. Similarly, a score of 1 indicates normal and 5 equals death.

For the evaluation of neuron death in the brain, the swine models were humanly euthanized with intravenous pentobarbital (50 mg/kg) at 96 hours and the hippocampus was acquired. A method of fluorescence TdT-Mediated dUTP Nick-End Labeling (TUNEL) (C1086, Beyotime Inc., Jiangsu, China) was used to measure cellular programmed death. Slides were observed under fluorescence microscope with a wavelength of 515–565 nm. TUNEL positive cells were calculated and compared with the ratio of apoptosis to survival cells in randomized 100 nucleuses between groups. All the histological tests were finished in the pathology teaching and research section of the Zhongshan School of Medicine (Sun Yat-sen University). The grouping of all samples was blinded to laboratory personnel.

### 2.5. Statistical Analysis

Data were presented as mean ± standard deviation (SD). Differences in compression depth and CPP between the two groups were analyzed by two-tailed Student's *t*-test for independent sample tests. A two-tailed Fisher's exact test was performed for rate comparison. A *P* value < 0.05 was regarded as statistically significant.

## 3. Results

### 3.1. Baseline Measurement

The baseline measurements of weight, hemodynamic status, and blood-gas analysis demonstrated no statistical significance between groups (Table 1).

### 3.2. Primary Resuscitation Outcome and CPR Quality

**Figure 1:**
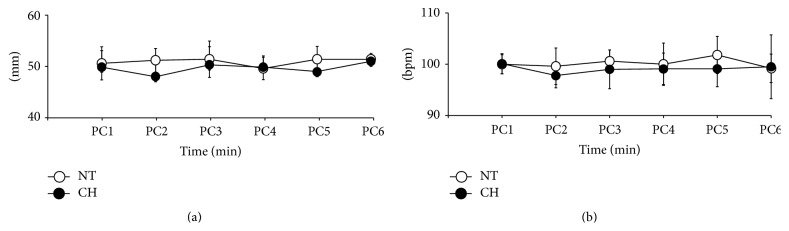
Compression qualities between groups during CPR. There were no differences of compression depth and rate between two groups.

During CPR, all animals in both groups received ROSC and consumed similar doses of epinephrine. Additional shocks were attempted in the animals in the NT group rather than in the CH group (2.17 ± 1.47 versus 1.71 ± 1.11, *P* = 0.552). However, there was no significant difference in the number of ROSC shocks and total shocks between groups. Although additional CPR time and limited first-shock success were performed in the NT group, there was no significant difference in the CH group (Table 2). The quality of CPR was the same between the two groups. There were no significant differences in compression depth and rate between the two groups during CPR (Figure 1).

### 3.3. Temperature Tendency

**Figure 2:**
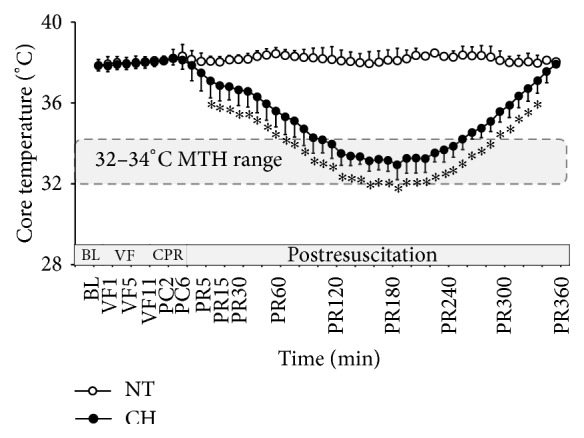
Tendency of core temperature (*T*_c_) between groups from baseline to postresuscitation. ^*∗*^ *P* < 0.05. BL = baseline, VF = ventricular fibrillation, CPR = cardiopulmonary resuscitation, MTH = mild therapeutic hypothermia, PR = postresuscitation.

The core temperature (*T*_c_) in the NT group was maintained at 37°C-38°C from baseline until 6 hours after resuscitation. *T*_c_ in the CH group decreased while MTH protocol began. The induction curve was steeper and the target MTH range of 32°C–34°C was reached within 2 hours. The maintaining duration was set until 4 hours after ROSC and followed by a 2-hour active rewarming phase. *T*_c_ from PR 10–340 mins displayed a significant difference compared to that in the NT sham group (*P* < 0.05). The actual cooling process was in accordance with the scheduled MTH protocol (Figure 2).

### 3.4. Myocardial Function

**Figure 3:**
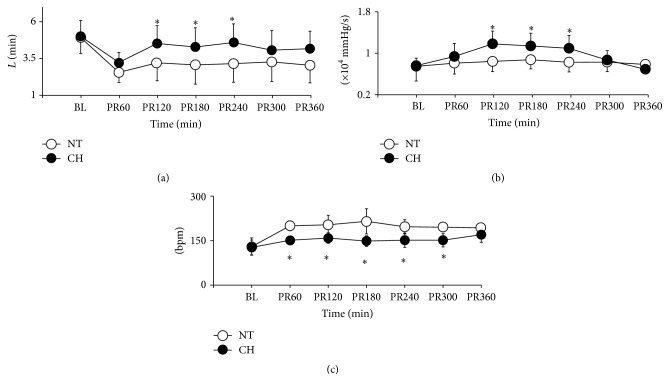
Cardiac output, dp/dt, and heart rate between groups. (a) Cardiac output; (b) *dp*/*dt*; (c) heart rate. ^*∗*^ *P* < 0.05. BL = baseline, PR = postresuscitation.

Myocardial function between groups was demonstrated in Figure 3. CO in the CH group decreased to nadir at 60 mins after resuscitation when compared to the NT group (3.23 ± 0.68 versus 2.56 ± 0.89, *P* = 0.10). Significant recoveries of CO were found at 2, 3, and 4 hours after ROSC during combined hypothermia when compared with the NT group (Figure 3(a)). The same results were found in myocardial contractive function of left ventricle. Cooling promoted myocardial contractility during the period of MTH (*P* < 0.05) and returned to baseline after rewarming (Figure 3(b)). It revealed that the heart beat at a slower pattern in the CH group was based on the physiological effect of hypothermia (Figure 3(c)).

### 3.5. Survival and Neurological Outcome

All animals in the CH group survived until 96 hours after ROSC (survival rate is 100%). Four animals in the NT group died within 24 hours and only three animals survived until 96 hours. Therefore, the animal survival time in the NT group was significantly less than in the CH group ([49.71 ± 43.65] hours versus [96.00 ± 0.00] hours, *P* = 0.03). For neurological function, significantly higher NDS and CPC (means worse neurological function) were observed in the NT group, when compared to the CH group until 96 hours after resuscitation (Table 3).

### 3.6. Neurons Histological Analysis

**Figure 4:**
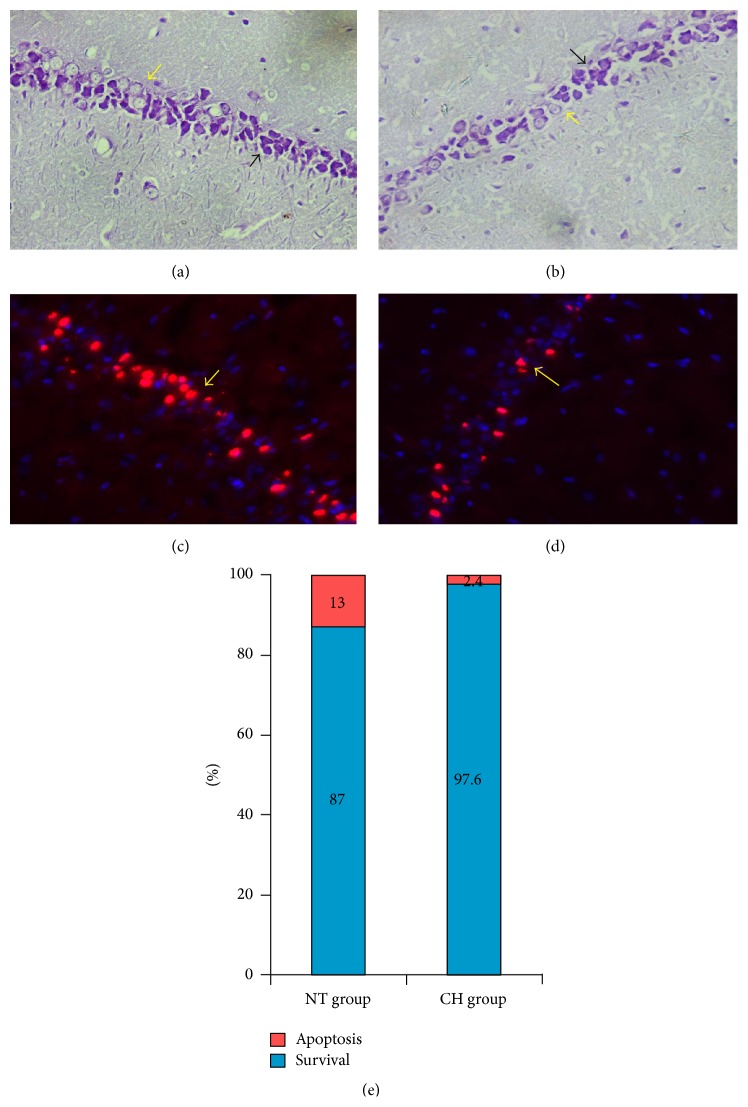
Neurons apoptosis in hippocampus between groups. Nissl staining of normal granule cell nucleus (black arrow) and dead neurons (yellow arrow) in NT group (a) and CH group (b); TUNEL of normal neuron nucleus (red label and yellow arrow) and TUNEL positive neurons (blue label) in NT group (c) and CH group (d). Percentage of TUNEL positive neurons and survival neurons between groups (Figure 4(e)).

Neurons histological analysis between groups was demonstrated in Figure 4. Additional vesicular cells were observed in the NT group, indicating that a large quantity of neurons died under normal ischemia/reperfusion (Figures 4(a) and 4(b)). Furthermore, an increase in TUNEL positive cells existed in the NT group when compared to the CH group (Figures 4(c) and 4(d)). It meant that MTH could rescue the vulnerable neurons from ischemia/reperfusion injury. Only 2.4% of the TUNEL positive cells were found in the CH group when compared with 13% in the NT group [(2.40 ± 0.55)% versus (13.00 ± 1.22)%, *P* < 0.05] (Figure 4(e)).

## 4. Discussion

In the present study we found that short-time combined cooling can preserve neurological and myocardial function, which results in better survival and neurological outcomes in a prolonged porcine model of cardiac arrest.

It had been strongly recommended that mild hypothermia should be applied in survivors of CA with a shockable rhythm. The evidence-based MTH protocol maintained the target temperature of 32°C–34°C for 12–24 hours. The goal of MTH was to maximize the preservation and minimize ischemia/reperfusion injuries. Generally, hypothermia was considered to play out over two time windows in two individual mechanisms: hypoxia-induced cellular dysfunction and reperfusion-induced cell death. MTH for 12–24 hours in the second time window had been proven to reduce mortality and improve the outcome in numerous patients and animals investigations [7, 10, 15]. However, studies indicated that the earlier the TTM is initiated in the first time window, the better the neurological outcome is [7–11]. A study of a rodent model of CA conducted by Abella et al. provided the evidence that intra-arrest cooling improved survival and neurological function after two hours of TTM [16]. Recently, Yannopoulos et al. also found that hypothermia induced rapidly in the first window provided beneficial outcomes in a rat model [10]. Our present study demonstrated that rapid cooling after ROSC undoubtedly offered an optimistic outcome in survival time and neurological recovery in this porcine model.

Different cooling strategies share different thermodynamic and physiologic principles in application. Transnasal cooling has the priority to cool the brain followed by systemic circulation and provides favorable outcomes in CPR [8]. However, it is too expensive to be popularized in the MTH protocol. Surface cooling is a traditional and effective technique but has the disadvantage of consuming more time to achieve target temperature and therefore leads to a longer duration and weak preservation. Ice-cold saline infusion is a popular cooling method. It is easy to apply and relatively safe when compared with other rapid cooling methods. However, the potential side effects of the fluid bonus limit its excessive application. Studies have demonstrated that cold fluids intravenous infusion could decrease coronary perfusion and increase incidence of cardiac rearrest and pulmonary edema [7, 10]. These undesired side effects might compromise the cooling outcome and interfere with MTH intentions in various environments. In our study, the cold saline volume was lower than the recommended level of 30–40 mL/kg [11]. When combined with surface cooling, a target temperature of 34°C can be successfully reached within two hours. It is feasible to achieve rapid cooling but difficult to avoid circulatory overload.

Compared with 12–24 hours of MTH in clinical practice, a relatively short duration of MTH in lab investigations demonstrates a beneficial outcome. In our study, the MTH protocol performed hypothermia with 4 hours of induction and maintained the target temperature for only 4 hours. We noted that myocardial function in the CH group was better preserved. Cardiac output was significantly higher in the CH group during maintenance of MTH (4.28 L/min–4.58 L/min versus 3.06 L/min–3.20 L/min, *P* < 0.05), as well as the *dp*/*dt*, which is an important parameter for evaluating left ventricular function. Furthermore, better neurological preservation after combined cooling was also observed in this shorter duration MTH study. Less necrosis and apoptosis of neurons in hippocampus were found in the CH group. In a research study with a gerbil model of global ischemia by Carroll and Beek, hypothermia initiated immediately following reperfusion must have a duration of 2 hours or more to be effective [17]. In this study, a short-time but prompt cooling could yield enough protection. Therefore, the clinical value of short duration MTH might be underestimated. Furthermore, an interesting study of a rat CPR model by Ye et al. compared three different durations of hypothermia after ROSC [13]. The study demonstrated a 2-hour cooling, rather than 5 or 8 hours, providing not only optimal ejection fraction and myocardial performance index (indicating better cardiac function) but also better survival and neurological outcomes. They concluded that a short duration of hypothermia therapy might supply CA patients with higher benefits but fewer complications and less cost.

The results in this study demonstrated that the neuron in hippocampus was sensitive to ischemia. Previous investigations had demonstrated that granule cells were prone to apoptosis in secondary reperfusion injury [18, 19]. This programmed cell death, initiated in the first 24 hours, may maximize in 2-3 days after ROSC [20–22]. In our hypothermia group, the number of apoptosis cells was significantly reduced and indicated that a shorter duration of MHT was effective on inhibition of apoptosis after resuscitation. We noticed the dimension of CPC and NDS had a weak association with central histological changes. Current methods of evaluating neurological function could not distinguish cognition dysfunction (a critical nerve reflex loop of hippocampus) from awaken status, which needs to be studied further.

There were several limitations in our study. We used healthy cardiac arrest swine models and ventricular fibrillation was induced electrically. Nevertheless, we chose 11 mins of a ventricular fibrillation model and the prolonged no-flow time of core organs could generate severe ischemic injury. The results were comparable with the real conditions that VF usually suffered from such as coronary artery occlusion or asphyxia. We only divided the animals into two groups; it is hard to tell if a short-time surface cooling can also yield the same positive outcomes as combined cooling. However, the previous study did not demonstrate if the single surface cooling method would yield such comparable good outcomes, especially in a porcine model of prolonged cardiac arrest [23]. Last, the rewarming rate in our study was 2°C/hour, which was much faster than the recommended level of 0.25°C–0.5°C/hour [5, 6]. Rapid rewarming might induce inflammation and increase cellular stress-response, which might eventually compromise cooling benefits. A rewarming rate at 0.5°C–1°C/hour may be suitable in a rodent model [24]. However, a recent retrospective cohort study supported that patients who need active rewarming after MTH did not have a higher risk for a poor outcome [25].

## 5. Conclusion

In this porcine model, short duration combined cooling with ice-cold saline infusion and surface cooling initiated after ROSC can improve survival and neurological outcomes.

## Figures and Tables

**Table 1:** Baseline measurement between two groups.

	NT (*n* = 7)	CH (*n* = 7)	*P* value
Body weight (kg)	34.17 ± 3.31	35.00 ± 3.00	*0.64*
Hemodynamic status			
MAP (mmHg)	113.70 ± 15.70	109.60 ± 10.45	*0.60*
CPP (mmHg)	98.80 ± 14.11	92.33 ± 13.42	*0.42*
LVDP (mmHg)	3.40 ± 2.76	3.00 ± 2.69	*0.80*
*dP*/*dt* (mmHg/s)	7471.17 ± 2823.22	7601.43 ± 1438.16	*0.92*
CO (L/min)	4.95 ± 0.95	5.00 ± 1.08	*0.93*
Blood-gas analysis			
*P*_ETCO_2__ (mmHg)	38.17 ± 2.48	40.29 ± 4.03	*0.27*
pH value	7.49 ± 0.03	7.52 ± 0.05	*0.20*
PaCO_2_ (mmHg)	36.38 ± 3.43	32.86 ± 3.50	*0.10*
Oxidation index (mmHg)	400.79 ± 83.05	458.50 ± 66.02	*0.20*
Core temperature (°C)	37.85 ± 0.36	37.84 ± 0.18	*0.97*
Lactate (mmol/L)	2.23 ± 2.11	2.91 ± 1.32	*0.51*

Values are expressed as mean ± SD.

**Table 2:** Comparison of CPR between two groups.

	NT (*n* = 7)	CH (*n* = 7)	*P* value
CPR time (min)	7.05 ± 1.05	6.29 ± 0.76	*0.17*
1st shock success (%)	50 ± 55	86 ± 38	*0.21*
ROSC shocks	1.50 ± 0.55	1.14 ± 0.38	*0.21*
Total shocks	2.17 ± 1.47	1.71 ± 1.11	*0.55*
ROSC (%)	100	100	*1*
Doses of epi. (mg)	1.50 ± 0.55	1.14 ± 0.36	*0.21*

Values are expressed as mean ± SD.

**Table 3:** Survival and neurological outcomes between groups.

	NT (*n* = 7)	CH (*n* = 7)	*P* value
Survival time (hrs)	49.71 ± 43.65	96.00 ± 0.00^*∗*^	*0.031*
96-hour survival (%)	42.86	100.00^*∗*^	*0.030*
CPC			
PR 24 h	4.00 ± 1.10	1.57 ± 0.53^*∗*^	*0.001*
PR 48 h	3.50 ± 1.76	1.00 ± 0.00^*∗*^	*0.017*
PR 72 h	3.33 ± 1.97	1.00 ± 0.00^*∗*^	*0.003*
PR 96 h	3.57 ± 1.90	1.00 ± 0.00^*∗*^	*0.011*
NDS			
PR 24 h	319.17 ± 99.42	79.29 ± 38.99^*∗*^	*0.001*
PR 48 h	269.17 ± 164.33	21.43 ± 26.73^*∗*^	*0.013*
PR 72 h	258.33 ± 184.87	6.67 ± 16.33^*∗*^	*0.020*
PR 96 h	255.83 ± 187.85	5.00 ± 12.25^*∗*^	*0.021*

^*∗*^ *P* < 0.05 versus NT group. PR means postresuscitation.
